# Child maltreatment and incident mental disorders in middle and older ages: a retrospective UK Biobank cohort study

**DOI:** 10.1016/j.lanepe.2021.100224

**Published:** 2021-09-27

**Authors:** John M Macpherson, Stuart R Gray, Patrick Ip, Marianne McCallum, Peter Hanlon, Paul Welsh, Ko Ling Chan, Frances S Mair, Carlos Celis-Morales, Helen Minnis, Jill P Pell, Frederick K Ho

**Affiliations:** a Institute of Health and Wellbeing, University of Glasgow, United Kingdom; b Institute of Cardiovascular & Medical Sciences, University of Glasgow, United Kingdom; c Department of Paediatrics and Adolescent Medicine, University of Hong Kong; d Department of Applied Social Sciences, Hong Kong Polytechnic University

**Keywords:** Child Abuse, Mental Health, Cohort Studies

## Abstract

**Background:**

Understanding the mental health consequences of child maltreatment at different life stages is important in accurately quantifying the burden of maltreatment. This study investigated the association between child maltreatment and incident mental disorders in middle and older age as well as the potential mediators and moderators.

**Methods:**

This is a retrospective cohort study of 56,082 participants from UK Biobank. Child maltreatment was recalled using the Childhood Trauma Screener. Incident mental disorders, including depressive, anxiety and affective disorders, behavioural syndromes, post-traumatic stress disorder (PTSD), schizophrenia, substance abuse, and dementia, after baseline assessment were ascertained through linkage to primary care records.

**Findings:**

There was a dose-response relationship between child maltreatment and mental disorder. Those who experienced three or more maltreatment types had the highest risk of all mental disorders (HR 1.85, 95% CI: 1.67-2.06) followed by those who experienced two (HR 1.48, 95% CI: 1.35-1.63) and then one (HR 1.26, 95% CI: 1.19-2.35). Child maltreatment was most strongly associated with PTSD (HR 1.59, 95% CI: 1.20-2.10 P=0.001). The excess risk was largely unexplained by the included mediators. The association between child maltreatment and all mental disorders were stronger among participants who binge drank (P_interaction_=0.003) or had few social visits (P_interaction_=0.003).

**Interpretation:**

The mental health consequence of child maltreatment could last decades, even among those who had no recorded mental disorders in early adulthood. In the absence of strong mediators, prevention of child maltreatment remains the priority.

**Funding:**

Wellcome Trust Institutional Strategic Support Fund

**Research in context**

**Evidence before this study**

We searched MEDLINE and PsycINFO, CINAHL for English language papers published between Jan 1, 2006, and Aug 13, 2020. We used a combination of search terms related to mental health and the mental disorders included and child maltreatment ([maltreat*] OR [abuse] OR [neglect]). There were numerous studies illustrating the associations between child maltreatment and childhood and early adulthood mental disorders. A meta-analysis study showed consistent associations between child maltreatment and mental disorders. However, many of the studies were subjected to various limitations, for example, retrospective cohort studies often used prevalent outcomes, which could substantially distort the exposure-outcome associations since prevalent mental disorders could affect maltreatment recall. While some studies had follow-up until early adulthood (<40 years), none have shown the association with incident mental disorders in middle and older age (i.e. no prior mental disorder diagnosis). There were also no studies that systematically investigated potential mediators and moderators.

**Added value of this study**

In this retrospective cohort study of 56,082 participants, child maltreatment was associated with incident mental disorder in middle and older age. No strong mediators were identified but the associations were stronger among participants who binge drank or had few social visits.

**Implications of all the available evidence**

Child maltreatment victims were at higher risk of incident mental disorders even if they were not diagnosed of any in early adulthood. Maltreatment victims who also binge drank or lacked social support were at greater risk of mental disorders. In the absence of strong mediators, prevention of child maltreatment remains the priority.

Alt-text: Unlabelled box

## 1 Introduction

Adverse childhood experiences (ACEs) are a global problem affecting 42% of children or adolescents in Europe and 58% in North America [1]. Child maltreatment, which comprises physical, emotional, and sexual abuse as well as neglect, is a major component of ACEs. Globally over 1 billion children and adolescents experience violence, and many more cases go unreported [2].

ACEs have been shown to lead to adverse mental disorders in adulthood, most notably depression, anxiety, and posttraumatic stress disorder (PTSD) [3,4] with a dose-response relationship. In a recent meta-analysis, participants with at least four ACEs had a higher risk of all adverse health outcomes; with strong associations shown for mental disorders, in particular depression (OR=4.4) and anxiety (OR=3.7) [5].

The associations between child maltreatment and mental disorders were consistently reported, again most notably on depression (OR=2.5) and anxiety (OR=1.7) [6]. However, many of the existing studies were subject to numerous limitations. For example, retrospective cohort studies often used prevalent outcomes, which could substantially distort the exposure-outcome associations since prevalent mental disorders could affect maltreatment recall [7]. The issue of reverse causation is particularly difficult to disentangle for early-onset mental disorders and there is a lack of evidence focusing on mental disorders that were firstly diagnosed in middle and older age.

It has been hypothesised that factors such as social support, resilience, and socio-economic status may mediate the relationship between ACEs, or child maltreatment, and mental health. However, the empirical evidence mostly focused on socio-economic status, which was also inconsistent [8,9]. There were also preliminary studies suggesting the potential mediating roles of systematic inflammation [10] and cardiovascular biomarkers [11].

The current study, therefore, aims to investigate the association between child maltreatment and the onset of a wide range of physician-diagnosed mental disorders in middle and older age, as well as potential mediating and moderating factors.

## 2 Methods

### 2.1 Study design and participants

This was a retrospective cohort study using data from UK Biobank. Child maltreatment was recalled by participants after they were recruited into the UK Biobank. Incident mental disorders were ascertained through record linkage after the baseline assessment. In this study, participants with prevalent/prior mental disorders at baseline assessment were excluded. This is to reduce recall bias and to focus on incident events in middle and older ages. The timeline of measurements is shown in Supplementary Figure 1.

UK Biobank recruited 502,493 participants aged 37-73 years (5.5% response rate) who were assessed at 22 centres across England, Scotland, and Wales between 2007 and 2010 as a general population-based cohort. UK Biobank received ethical approval from the North West Multi-Centre Research Ethics Committee (REC reference: 11/NW/03820).

Recruitment was conducted by sending letters to home addresses obtained from national health records, and follow-up data were obtained via linkage to routine health records, web-based questionnaires and repeat assessment clinics attended by a sub-group (https://www.ukbiobank.ac.uk/). At the baseline assessment clinic, participants completed a self-administered questionnaire on general health and a more in-depth face to face interview on medical history. Self-reported variables included ethnicity, education level, sleep duration, television viewing time, smoking status, and alcohol intake. Deprivation level was measured via the Townsend area deprivation index which sums a score derived from assets, income, and household factors applied to postcode of residence [12]. The International Physical Activity Questionnaire (IPAQ) was used to assess physical activity levels [13].

Following the baseline interviews, a series of measurements, including height, weight, and blood pressure, were collected along with urine and blood samples. Body mass index (BMI) was derived from weight/height^2^ measured using standardized equipment and used to classify individuals as underweight (<18.5 kg/m^2^), normal weight (18.5 to 24.9 kg/m^2^), overweight (25 to 29.9 kg/m^2^), or obese (>30.0 kg/m^2^). Biomarkers were measured at a dedicated central laboratory between 2014 and 2017. In this study, we have selected triglyceride (TG), a lipid found to be associated with mood disorders [14], and C-reactive protein (CRP), a marker of systemic inflammation and indicator of a stress response [15]. Details of these measures and assay performances can be found online in the UK Biobank showcase and protocol [16].

### 2.2 Child maltreatment

Child maltreatment was assessed through a web-based questionnaire conducted in August 2017 [17]. Overall 339,229 participants who provided an email address were invited and approximately half (*n* = 157,348) of participants completed the online questionnaire. Respondents were generally younger, more likely to be female and white, and had healthier lifestyles compared with non-respondents [18]. The web-based questionnaire included the Childhood Trauma Screener (CTS) [19], a shortened version of the Childhood Trauma Questionnaire (CTQ) [20]. It consists of a 5-point Likert scale for each of five types of child maltreatment, physical abuse, physical neglect, emotional abuse, emotional neglect, and sexual abuse, and has been validated against the CTQ with good overall correlation (*r* = 0.88) and satisfactory type-specific correlations (*r* = 0.55–0.87) [21]. The CTQ is a widely used instrument for measuring child maltreatment and has been validated against actual records of abuse and neglect [19,22]. The threshold values on the Likert scale derived from a validation study [19] were used to define the presence or absence of each type of child maltreatment. In this study, the primary exposure variable was the number of types of child maltreatment, and was categorised as 0, 1, 2, and ≥3 as there were not sufficient number of events to analyse participants with 4 or 5 types of maltreatment separately.

### 2.3 Mental disorders

Mental disorders were ascertained through individual-level record linkage to primary care records available for 45% of the UK Biobank cohort until May 2017 for Scotland, September 2017 for Wales, and August 2017 for England. The detailed linkage procedures relating to primary care records are available online (http://biobank.ndph.ox.ac.uk/showcase/showcase/docs/primary_care_data.pdf). READ codes in the primary care record were mapped to ICD-10 codes. The outcomes in this study included: all mental disorders (ICD-10 F01-F99), dementia (F01-F03), substances abuse (F10-F19), including alcohol-related, disorders, schizophrenia (F20-F29), affective disorder (F30-F39), depression disorder (F32-F33), anxiety disorder (F40-F48), PTSD (F43.1), and behavioural syndrome (F50-F59) encompassing eating and sleep disorders, as well as syndromes associated with physiological disturbances and physical factors.

### 2.4 Statistical analyses

Cox proportional hazard models were used to analyse the associations between child maltreatment and incident mental disorders. The results are reported as hazard ratios (HRs) with 95% confidence intervals (CIs). In the primary analysis we only included participants who did not have any mental disorder diagnosis at or before baseline assessment. This can reduce reverse causation because prevalent mental disorders could affect child maltreatment recall [7]. The models were adjusted for age at baseline assessment, sex, and ethnicity as potential confounders. Deprivation and education could be the consequence of child maltreatment (mediator) or affecting child maltreatment recall (confounder), but were primarily hypothesised to be mediators in the analysis. The hypothetical causal assumption is shown in Supplementary Figure 2. Since excluding participants who had mental disorder diagnosis prior to baseline assessment may induce selection bias, we conducted sensitivity analyses including all participants with complete data. In these sensitivity analyses, follow-up was assumed to start when they were at the age of 30 years and all mental disorder diagnoses after that were included.

Moderation analyses were conducted by: sex, age group (38–50, 51–60, and 61–72 years; approximate tertiles), education level (with vs. without university degree), and area-based deprivation index (≥ vs. < median), physical activity (<600 vs ≥600 MET-min/week), obesity (BMI ≥30kg/m^2^ vs <30kg/m^2^), alcohol drinking (≤14 vs >14 units/week), binge drinking (binge drinker [average drinking unit per occasion >6 for female and >8 for male] vs non-binger drinker), ability to confide (never/almost never vs sometimes or more), and frequency of social visits (never/almost never vs sometimes or more), and household factors (household size, living with partner, living with children, living with relatives, living with unrelated person/people). We selected these factors because age could affect the reporting of maltreatment, sex could affect the type of maltreatment, and socioeconomic and lifestyle factors might influence the long-term effects of maltreatment experiences. Both stratified (sub-group) and interaction analyses were conducted for mental disorders, depression disorder, anxiety disorder, and behavioural syndrome as these outcomes had sufficient events for subgroup analyses. We also conducted sensitivity analyses including all mental disorder diagnoses for the moderation analysis of sex, age group, and education level as these factors should be the same or very similar between age of 30 and the baseline assessment.

We studied five groups of potential mediators: PA and TV (physical activity >600 MET-min/week [binary variable], time spent watching television [continuous variable]), smoking and drinking (any smoking and alcohol drinking >14 units a week [binary variable]), social factors (able to confide and social visit frequency [binary variables]), CVD risk factors (obesity and blood pressure [continuous variable]), and CRP and TG (continuous variable). All potential mediators were selected because of their potential associations with both child maltreatment and mental health. These groups of mediators were adjusted for in the Cox models to examine whether, and to what extent, the HRs between child maltreatment and mental disorders were attenuated.

Formal mediation analysis based on counterfactual framework was then conducted [23]. Counterfactual framework defines direct (non-mediated) and indirect (mediated) effects and are more robust against various limitations of traditional adjustment-based mediation analysis, such as mediator-outcome confounding affected by exposure [24]. To reduce multicollinearity and overadjustment, the potential mediators were selected using a stepwise approach. Firstly, mental disorder was regressed by child maltreatment and all potential mediators and confounders in a Weibull regression model with robust standard errors. Weibull regression was chosen because of its superior statistical properties in mediation analysis [25]. Potential mediators were then selected based on their associations with mental disorder after mutual adjustments. The selected potential mediators were then regressed by child maltreatment count and other covariates (mediator model) in either logistic (for binary mediators) or linear (for other mediators) models adjusting for other mediators and confounders. The outcome and mediator models were then combined to compute the mediation proportions based on natural indirect effect (NIE) divided by total effect (TE) for each participant which was then averaged. Quasi-Bayesian estimation with 1,000 iterations were used for estimating the 95% CI and p-values of the NIE and TE.

Analyses were conducted in R version 4.0.2 using packages *survival* and *mediation*.

### 2.5 Role of funding sources

The funder has no roles in study design, data collection, data analysis, interpretation, and writing of the report.

## 3 Results

Of the 502,493 UK Biobank participants, 152,911 had complete data on child maltreatment and sociodemographic covariates (Supplementary Figure 3). Of these, 68,338 had been linked to primary care records which revealed that 12,256 had a mental disorder prior to the baseline assessment. These were excluded in the main analysis to account only for new onset of mental disorders resulting in a study population of 56,082 participants; mean [SD] age 55.49 [7.74] years and 59.86% female. At least one type of child maltreatment was reported by 31.10% (17,443) of participants; 19.75% (11,078), 6.92% (3,879) and 4.43% (2,486) of participants reported 1, 2, and ≥3 types respectively. The most frequently reported maltreatment type was emotional neglect (20.2%), and the least was physical neglect (5.21%). Those with a higher number of reported child maltreatments were: younger; more likely to be deprived, a smoker and obese; watched more TV; and were less likely to be physically active; have a university/college degree; or be able to confide (Table 1). Overall, 5,595 (9.98%) participants developed a mental disorder over the follow-up period. Anxiety disorder was the most common (n=1,865 [3.33%]) followed by behavioural syndrome (n=1,420 [2.53%]), and affective disorder (n=1,304 [2.33%]), including depression disorder (n=1,229 [2.19%]).

**Table 1:** Participants characteristics by number of child maltreatment types

	0	1	2	3+
Total n	38,639	11,078	3,879	2,486
Mean (SD) age, years	56.19 (7.69)	55.91 (7.75)	55.35 (7.73)	54.50 (7.80)
Male	17209 (44.54)	4956 (44.74)	1526 (39.34)	786 (31.62)
Ethnicity				
White	37938 (98.19)	10745 (96.99)	3728 (96.11)	2332 (93.81)
South Asian	251 (0.65)	123 (1.11)	39 (1.01)	39 (1.57)
Black	124 (0.32)	60 (0.54)	43 (1.11)	46 (1.85)
Chinese	62 (0.16)	32 (0.29)	13 (0.34)	14 (0.56)
Mixed	122 (0.32)	60 (0.54)	29 (0.75)	28 (1.13)
Others	142 (0.37)	58 (0.52)	27 (0.70)	27 (1.09)
Less deprived (index < median)	20149 (52.15)	5212 (47.05)	1679 (43.28)	1000 (40.23)
Education attainment	18285 (47.32)	4983 (44.98)	1675 (43.18)	1036 (41.67)
Smoking				
Never	24368 (63.17)	6471 (58.51)	2087 (53.98)	1215 (49.01)
Previous	12710 (32.95)	4043 (36.56)	1539 (39.81)	1094 (44.13)
Current	1499 (3.89)	546 (4.94)	240 (6.21)	170 (6.86)
Alcohol drinking > 14 units/week	20149 (52.15)	5212 (47.05)	1679 (43.28)	1000 (40.23)
Physical activity > 600 MET-minutes/week	18285 (47.32)	4983 (44.98)	1675 (43.18)	1036 (41.67)
Mean (SD) TV viewing/day	2.45 (1.36)	2.48 (1.43)	2.53 (1.47)	2.56 (1.52)
Able to confide	33727 (87.29)	9196 (83.01)	3137 (80.87)	1986 (79.89)
Have social visits	38252 (99.00)	10893 (98.33)	3790 (97.71)	2424 (97.51)
Household size	2.47 (1.17)	2.41 (1.13)	2.37 (1.13)	2.40 (1.17)
Living with partner	30403 (78.68)	8206 (74.07)	2751 (70.92)	1711 (68.83)
Living with children	13920 (36.03)	3859 (34.83)	1361 (35.09)	902 (36.28)
Living with other relatives	1125 (2.91)	323 (2.92)	97 (2.50)	69 (2.78)
Living with non-relatives	532 (1.38)	218 (1.97)	80 (2.06)	65 (2.61)
BMI categories				
Underweight	195 (0.51)	62 (0.56)	20 (0.52)	15 (0.60)
Normal	15287 (39.63)	4107 (37.17)	1368 (35.30)	814 (32.82)
Overweight	16022 (41.54)	4584 (41.49)	1615 (41.68)	976 (39.35)
Obese	7070 (18.33)	2296 (20.78)	872 (22.50)	675 (27.22)
Central obesity	15930 (41.28)	4728 (42.78)	1633 (42.11)	1036 (41.76)
Mean (SD) systolic blood pressure, mmHg	137.21 (18.07)	136.56 (18.22)	135.58 (18.10)	134.93 (18.58)
Mean (SD) handgrip strength, kg	31.63 (10.72)	31.56 (10.83)	30.74 (10.92)	29.48 (10.54)
Mean (SD) c-reactive protein, mg/L	2.19 (4.16)	2.31 (4.53)	2.44 (4.80)	2.82 (5.58)
Mean (SD) triglyceride, mmol/L	1.64 (0.96)	1.67 (0.96)	1.67 (1.03)	1.67 (1.00)

Abbreviations: N- sample size, SD- standard deviation, GCSE- General Certificate of Secondary Education, SE- secondary education, NVQ- National Vocational Qualification, HND- Higher National Diploma, HNC- Higher National Certificate, MET- Metabolic Equivalent for Task,

**Fig. 1:**
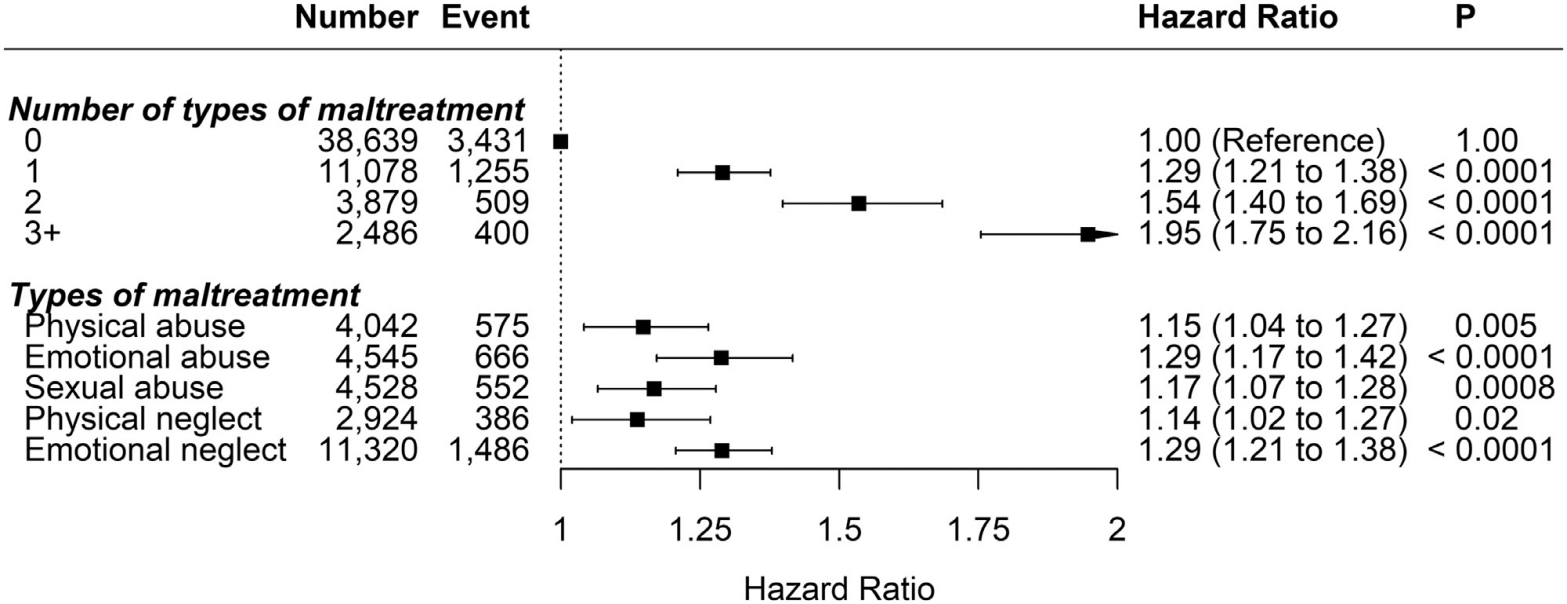
Association between child maltreatment and all mental disorders Adjusted for age, sex, and ethnicity.

Child maltreatment was associated with overall mental disorders with evidence of a dose-response relationship (Fig. 1). Compared with participants who did not report any of the included maltreatment type, those who reported three or more types of maltreatment had the highest risk (HR 1.95, 95% CI: 1.75-2.16), followed by those who experienced two (HR 1.54, 95% CI: 1.40-1.69) then one (HR 1.29, 95% CI: 1.21-2.38). When mutually adjusted, emotional abuse (HR 1.29, 95% CI: 1.17-1.42) and neglect (HR 1.29, 95% CI: 1.21-1.38) had the stronger independent associations with all mental disorders, followed by sexual (HR 1.17, 95% CI: 1.07-1.28) and physical (HR 1.15, 95% CI: 1.04-1.27) abuse, even though the 95% CIs overlapped.

**Fig. 2:**
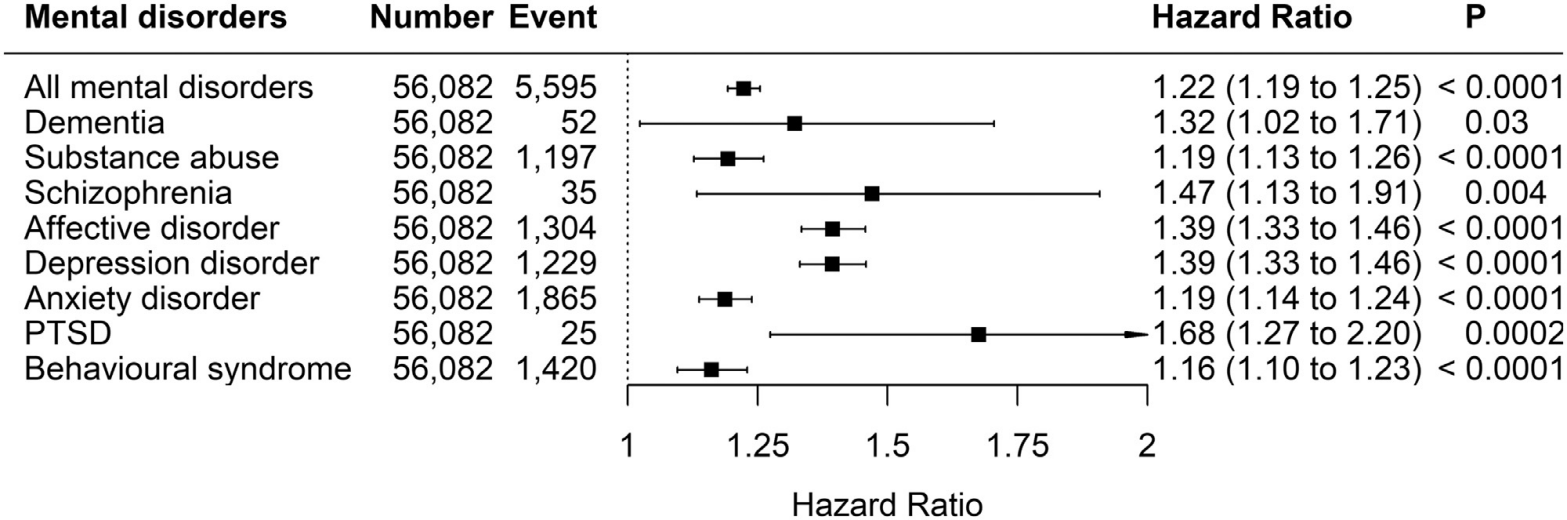
Association between number of maltreatment types and mental disorders Adjusted for age, sex, and ethnicity. The HRs correspond to the increase in risk for each additional types of child maltreatment.

The associations between number of child maltreatment types and mental disorders are shown in Fig. 2. Child maltreatment was associated with all mental disorders. The association was strongest for PTSD (HR 1.68, 95% CI: 1.27-2.20), and weakest for behavioural syndrome (HR 1.16, 95% CI:1.10-1.23), substance abuse (HR 1.19, 95% CI: 1.13-1.26), and anxiety disorder (HR 1.19, 95% CI: 1.14-1.24).

**Fig. 3:**
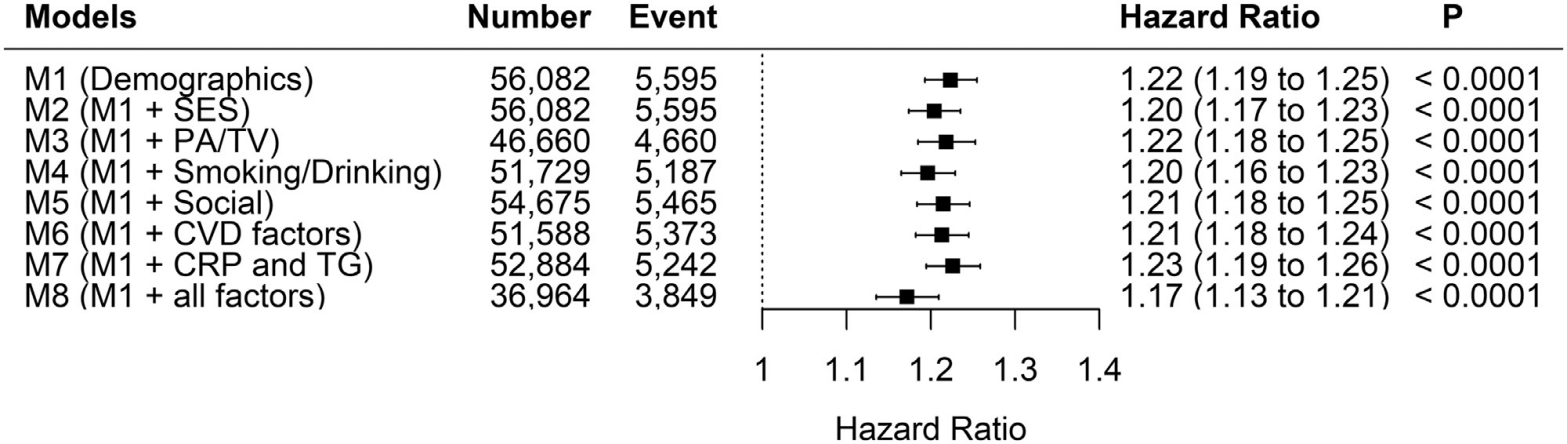
Association between number of child maltreatment types and all mental disorders by adjustment models M1: Adjusted for age, sex, and ethnicity M2: M1 and additionally deprivation index, and education attainment M3: M1 and additionally total physical activity level and television viewing M4: M1 and additionally smoking status and units of alcohol drinking M5: M1 and additionally able to confide and social visit frequency M6: M1 and additionally handgrip strength and systolic blood pressure M7: M1 and additionally adjusted for C-Reactive Protein and Triglyceride M8: Adjusted for all factors listed The HRs correspond to the increase in risk for each additional types of child maltreatment.

Results following adjustment for potential mediators are contained in Fig. 3. Child maltreatment was significantly associated with overall mental disorders even after adjustment for mediators. The HR was slightly attenuated after adjusting for all mediators (from HR=1.22 to HR=1.16). Table 2 shows the summary of the formal mediation analysis. University degree, TV viewing, able to confide, and systolic blood pressure were significant mediators even though they only mediated small proportions of the excess risk due to child maltreatment. Supplementary Table 1 shows the estimated TE and NIE of the mediators.

**Table 2:** Summary of mediation analysis

	Association with all mental disorders*HR (95% CI)	Association with child maltreatment†β or OR^‡^ (95% CI)	% mediated
University degree^‡^	0.86 (0.80, 0.92)	0.88 (0.86, 0.90)	0.9
Area-based deprivation^‡^	0.95 (0.89, 1.02)	0.96 (0.94, 0.99)	-
MET-min per week	0.99 (0.96, 1.02)	0.04 (0.03, 0.05)	-
Hours of TV viewing	1.06 (1.02, 1.09)	0.02 (0.01, 0.03)	0.4
Units of alcohol intake	1.06 (1.03, 1.09)	-0.01 (-0.02, 0.00)	-
Able to confide^‡^	0.89 (0.82, 0.97)	0.80 (0.78, 0.83)	8.0
Social visits	0.81 (0.63, 1.04)	0.00 (-0.01, 0.00)	-
Obesity^‡^	1.06 (0.98, 1.16)	1.10 (1.06, 1.13)	-
Central obesity^‡^	1.15 (1.06, 1.24)	1.05 (1.02, 1.08)	0.9
Systolic blood pressure	0.93 (0.89, 0.96)	-0.02 (-0.03, -0.01)	1.2
CRP	0.99 (0.96, 1.02)	0.02 (0.01, 0.03)	-
Triglycerides	1.03 (1.00, 1.06)	0.01 (0.00, 0.02)	-

⁎ Cox regression models with potential mediators as independent variables.

† Linear (for continuous mediators) and logistic (for binary mediators^‡^) models with potential mediators as dependent variables. All analyses adjusted for each other and for child maltreatment, age, sex, and ethnicity

**Fig. 4:**
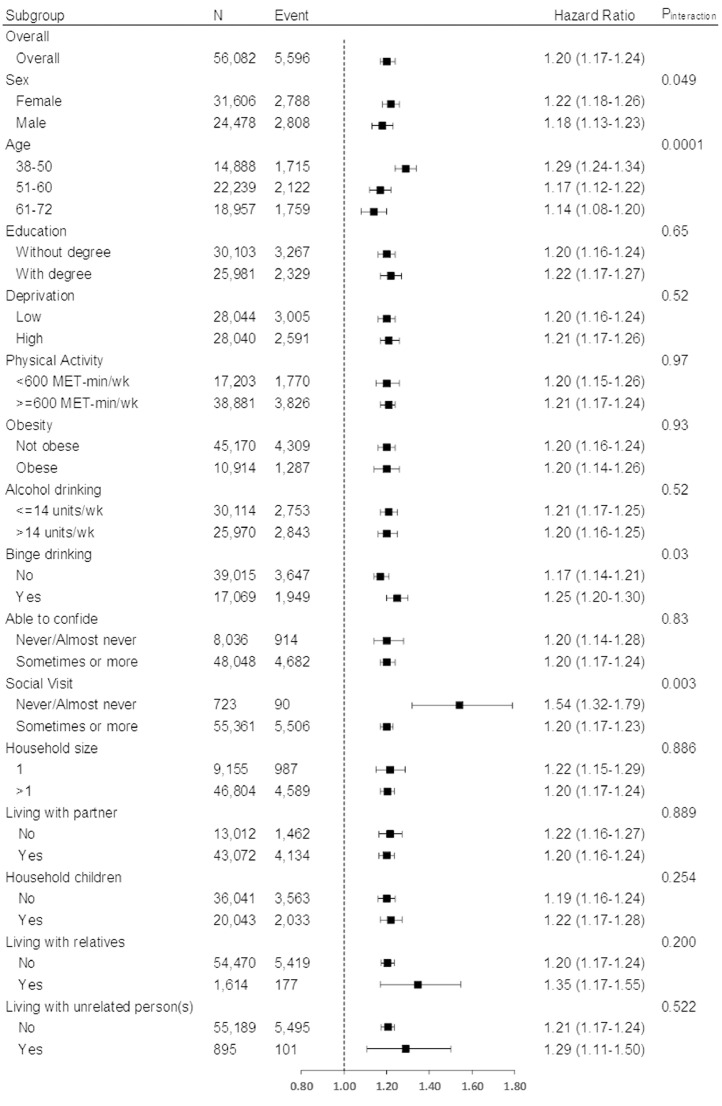
Association between number of child maltreatment types and all mental disorders by population subgroups Adjusted for age, sex, ethnicity, deprivation index, and education attainment. The HRs correspond to the increase in risk for each additional types of child maltreatment.

Fig. 4 shows the associations with overall mental disorders by sociodemographic, lifestyle, and household subgroups. The association between child maltreatment and mental disorders was stronger among participants who were female (P_interaction_=0.049), younger (P_interaction_=0.0001), binge drank (P_interaction_=0.03), and had fewer social visits (P_interaction_=0.003). No interactions reached statistical significance for depression disorder (Supplementary Figure 4). The associations with anxiety disorder were stronger among participants who were male (P_interaction_=0.02), younger (P_interaction_=0.01), undertook more physical activity (P_interaction_=0.0005), and had fewer social visits (P_interaction_=0.01) (Supplementary Figure 5). Binge drinking (P_interaction_=0.003) was the only significant moderator of the association with behavioural syndrome (Supplementary Figure 6). The subgroup of participants who never or almost never received a social visit had stronger associations between child maltreatment and three out of four outcomes (except behavioural syndrome), with an average 65% higher HR estimate than participants who received more frequent social visits.

The results for the sensitivity analyses which included participants who had previously been diagnosed with a mental disorder (n=12,256) are included in Supplementary Figures 7-9. Supplementary Figure 7 is generally consistent with Fig. 1 with the largest difference being a stronger association with mental disorders following 3 or more types of maltreatment (HR 2.04, 95% CI: 1.93-2.16 compared with HR 1.95, 95% CI: 1.75-2.16). The association between number of maltreatment types and different mental disorders (Fig. 2) shows similar results with the addition of participants with previous mental disorder diagnosis (Supplementary Figure 8). Mental disorders by population subgroup show an overall small increase with inclusion of those with previous mental disorder diagnoses (Supplementary Figure 9). Age shows a small increase in association for the two older age categories 51-60 and 61-72 but not for the 38-50 category.

## 4 Discussion

The current study has demonstrated a potentially important association between child maltreatment and mental disorders that spanned affective disorder (including depression), anxiety disorder (including PTSD), behavioural syndrome, substance abuse, and schizophrenia. Of the different types of maltreatment, emotional abuse and neglect had the stronger associations with long-term mental disorders. The associations were generally consistent whether we included only participants who were free from mental disorder diagnosis in middle or older age or included all participants including those with a previous diagnosis. This indicates that the potential mental health consequences of child maltreatment are apparent 20-70 years after the occurrence, even among individuals with no history of mental disorders in early adulthood. The long-term effects of child maltreatment were only partially mediated by lifestyle or biological factors. The associations were consistent irrespective of sex, level of education, deprivation, and physical activity, and presence of obesity, but were stronger amongst binge drinkers and people with few social visits. This could potentially be used to identify patients who are at the highest risk to develop mental disorders (e.g. those who binge drink and experienced child maltreatment). Whilst the potential harms and benefits of routine ACE/child maltreatment screening are still to be explored [26], clinicians could consider preventive measures if the patients are known to have history of child maltreatment and other compounding factors.

### 4.1 Comparison with existing literature

This study supports findings from the existing literature that demonstrated a clear association between ACE/child maltreatment and psychiatric outcomes in adulthood. A meta-analysis has identified associations of ACEs with depression and anxiety with strong effect sizes (ORs 4.4 and 3.7) [5], similarly in another meta-analysis on child maltreatment (ORs 2.5 and 1.7) [6]. However it should be noted that many of the studies included were subject to various biases and therefore the pooled effect sizes may be inflated.

A prospective study with 21-year follow up showed a consistent relationship between substantiated child maltreatment and mental disorders in early adulthood [4]. Consistent with our study and others [27], the authors found that emotional abuse and neglect were particularly harmful to subsequent mental health and PTSD was the outcome most strongly associated with all forms of abuse. The reasons behind the stronger association of emotional abuse are still to be explored it could be related to how the abuses were reported in this study. Physical abuse was reported as being hit so hard that it left the participants with bruises or marks in childhood, while emotional abuse was reported as being hated. The former could be, historically, a form of disciplinary method but the latter is most likely malicious. Further study should explore on this. Our study expands the existing evidence by showing that the association between child maltreatment and mental disorders continues into middle and older age, even if there were no known mental problems in childhood and early adulthood.

We found that good social support, as measured through frequent social visits, could modify the association between child maltreatment and mental disorders. However, we should note that reverse causation is possible as social isolation is a precursor to mental disorders. Similarly a prospective cohort study [28] found social support to be a significant effect modifier for child maltreatment and depression and anxiety. We found frequency of social visits to be a moderator of anxiety following maltreatment, however, it did not reach statistical significance for depression contrary to other literature [28,29]. It should be noted that there are many forms of social support and frequency of social visits encompasses just one; other forms such as trusted adult support through childhood, perceived social support, and social connectedness have also been shown to be important buffering factors [8,30,31].

Even though the ability to confide was the strongest mediator identified in this study, it only mediated 8% of the excess risk. It should be noted that the included social factors could not completely capture social support. Indeed, Sperry & Widom [28] tested the mediating effects of four types of social support on depression and anxiety following maltreatment and found total social support to be the strongest mediator reducing anxiety and depression to non-significance. However, they did not get this effect when the specific types of social support were considered individually suggesting overall social support is more important than its sub-types. Social factors are multidimensional including aspects like belonging, tangible support, appraisal, and self-esteem support and we may not have sufficient measurements to capture them comprehensively.

Our analysis found age to be a significant moderator of the association between child maltreatment and mental disorders with younger participants demonstrating a stronger association. This pattern remained even after inclusion of those with previous mental disorder diagnoses suggesting onset of mental disorders is most common with younger adults but persists into older age. Adolescence and early adulthood is thought to be a critical period for determining health in later adulthood [32] with mental disorders more common during this time than any other life stage [33]. However, the youngest UK Biobank participants included in our study were already 38 years of age. Further studies are needed to explore whether our observed interaction with age is due to a birth cohort effect, a weakening association over the life-course, or because maltreatment victims were more likely to die early [34].

It has been suggested that binge drinking could lie on the causal pathway from child abuse to mental disorders [35]. Previous studies have reported that childhood verbal abuse was associated with adult binge drinking in both men and women across five US states [36] and that child maltreatment was associated with both depression and binge drinking, independent of household challenges [37]. Our study adds to the existing evidence by demonstrating that binge drinking is an effect modifier, rather than a mediator. We demonstrated that those who reported binge drinking were more vulnerable to any mental disorders (P_interaction_=0.03) and behavioural syndrome (P_interaction_=0.003) following child maltreatment. Therefore, maltreatment victims who also have drinking problems represent a particularly vulnerable subgroup for mental illness and should be supported.

### 4.2 Limitations

Firstly, the UK Biobank does not have data on ACEs except for child maltreatment which was recalled by participants rather than captured prospectively. This is a common limitation of studies of childhood experiences with long duration of follow-up and may be subject to reverse causation. Also, retrospective measures may be liable to underreporting which could underestimate the associations [8,38,39]. Secondly although the number and type of maltreatments were recorded, severity, timing, duration, and frequency were not. Thirdly, other mediating and moderating factors such as neurodevelopmental disorders, resilience, and emotional dysregulation could not be analysed as they were not assessed in UK Biobank, and that the included mediators were only measured once in the baseline assessments. The low mediation proportions could be, partly, due nondifferential misclassification bias from the measurements of the mediators, as well as the omission of interactions between mediators. Another limitation is the potential for reverse causality with some moderators, in particular social factors and binge drinking. The primary outcome (all mental disorders) included alcohol-related disorders and therefore the moderation analysis for that could be overestimated. The UK Biobank cohort was not representative of the UK general population as they were more educated, less deprived, and had a healthier lifestyle (e.g. more physical activity) but the exposure-outcome associations were found consistent with population representative studies [40]. It should, however, be noted that only about 10% of the UK Biobank participants was included in this analysis. This might induce collider bias, inflating the associations, and that the results from this study might not be applicable for early adulthood onset mental disorders. Incident mental disorders were defined from after the baseline follow-up even though the child maltreatment were recalled after baseline assessment. The mental disorders were ascertained using primary care records, available in 45% of the participants. While the availability of primary care data is due to differences in recording system and should not cause differential bias, the reliance on primary care data may systematically omit some cases, particularly those with subclinical symptoms or those who refused or delayed to seek help [41]. There are also other mental disorders and manifestations of mental health conditions such as self-harm and suicide attempts which might not comprehensively captured in the primary care data and should be an area for future research. Whether child maltreatment may affect help seeking behaviours is not well understood. This could cause reverse causation and exaggerate the HRs.

### 4.3 Conclusions

Child maltreatment was associated with a wide range of mental disorders diagnosed for the first time in middle and older age. As no strong mediators were identified, prevention of child maltreatment should be prioritised to reduce maltreatment-related mental health burdens. Individuals maltreated in childhood who also binge drink and/or have weak social connectedness were at greater risk of developing mental disorders and could potentially be targeted for future interventions.

## Author contributions

John Macpherson analysed the data and drafted the manuscript. Stuart Gray, Patrick Ip, Marianne McCallum, Peter Hanlon, Paul Welsh, Ko Ling Chan, Frances Mair, Carlos Celis-Morales interpreted the data and critically revised the manuscript. Helen Minnis and Jill Pell conceptualised the study, interpreted the data, and critically revised the manuscript. Frederick Ho conceptualised the study, analysed the data, and drafted the manuscript.

## Declaration of interests

The authors declared no potential conflicts of interest.

## Appendix Supplementary materials

Image, application 1

Image, application 2

## Footnotes

Supplementary material associated with this article can be found in the online version at doi:10.1016/j.lanepe.2021.100224.
