# Supplementary material for: Child maltreatment and incident mental disorders in middle and older ages: a retrospective UK Biobank cohort study
Source: Lancet Reg Health Eur. 2021 Sep 27;11:100224. doi: 10.1016/j.lanepe.2021.100224 (PMC8642708; doi:10.1016/j.lanepe.2021.100224)
Supplement: Supplementary file 1 [file mmc1.docx]

**Supplementary Table 1**. Total and natural indirect effects from mediation analysis

**Supplementary Figure 1**. Timeline of measurements

**Supplementary Figure 2**. Conceptual causal pathway between child maltreatment and mental disorders

**Supplementary Figure 3**. Participant flow diagram

**Supplementary Figure 4**. Association between number of child maltreatment types and depression disorder by population subgroups

**Supplementary Figure 5**. Association between number of child maltreatment types and anxiety disorder by population subgroups

**Supplementary Figure 6**. Association between number of child maltreatment types and behavioural syndrome by population subgroups

**Supplementary figure 7.**  Association between child maltreatment and all mental health disorders including those with previous mental health diagnoses (n=68338)

**Supplementary figure 8.** Association between number of maltreatment types and mental outcomes disorders including those with previous mental health diagnoses (n=68338)

**Supplementary figure 9.**  Association between number of child maltreatment types and all mental disorders by subgroups disorders including those with previous mental health diagnoses (n=68338)
