# Supplementary material for: Child maltreatment and incident mental disorders in middle and older ages: a retrospective UK Biobank cohort study
Source: Lancet Reg Health Eur. 2021 Sep 27;11:100224. doi: 10.1016/j.lanepe.2021.100224 (PMC8642708; doi:10.1016/j.lanepe.2021.100224)
Supplement: Supplementary file 2 [file mmc2.docx]

Child maltreatment and incident mental disorders in middle and older ages: a retrospective UK Biobank cohort study

John M Macpherson^1^, Stuart R Gray^2^, Patrick Ip^3^, Marianne McCallum^1^, Peter Hanlon^1^,
Paul Welsh^2^, Ko Ling Chan^4^, Frances Mair^1^, Carlos Celis-Morales^1,2^,
Helen Minnis^1*^, Jill P Pell^1*^, Frederick K Ho^1*^

1. Institute of Health and Wellbeing, University of Glasgow, United Kingdom
2. Institute of Cardiovascular & Medical Sciences, University of Glasgow, United Kingdom
3. Department of Paediatrics and Adolescent Medicine, University of Hong Kong
4. Department of Applied Social Sciences, Hong Kong Polytechnic University

* Co-senior authors

**Corresponding author**

Frederick Ho

Institute of Health and Wellbeing

University of Glasgow

1 Lilybank Gardens

Glasgow G12 8RZ

United Kingdom

Email: [Frederick.Ho@glasgow.ac.uk](mailto:Frederick.Ho@glasgow.ac.uk)

Supplementary materials

**Supplementary Table 1**. Total and natural indirect effects from mediation analysis

|  |  | **95% CI** | |
| --- | --- | --- | --- |
|  | **Coefficient** | **Lower** | **Upper** |
| Total effect | -0.18 | -0.21 | -0.15 |
| Natural indirect effect |  |  |  |
| University degree | -0.0017 | -0.0027 | -0.0009 |
| Hours of TV viewing | -0.0007 | -0.0014 | -0.0002 |
| Able to confide | -0.0156 | -0.0198 | -0.0120 |
| Central obesity | -0.0015 | -0.0032 | 0.00003 |
| Systolic blood pressure | -0.0023 | -0.0037 | -0.0010 |

Coefficients are from Weibull regression on log scale. A negative coefficient indicates an association with earlier time-to-event (i.e. higher risk).

**Supplementary Figure 1**. Timeline of measurements

UK Biobank recruitment and baseline assessment

Recall of maltreatment in childhood

Incident mental disorders

in primary care data

No prior records of

mental disorders

2007-2010

2017

2007-2017

Timeline is not drawn to scale.

**Supplementary Figure 2**. Conceptual causal pathway between child maltreatment and mental disorders

Lifestyle factors

Incident mental disorder

Social factors

CVD factors

Inflammation

Child maltreatment

Prevalent/prior mental disorder

Adulthood SES

causing

causing

affecting

recall

affecting

recall

Childhood family SES

Some pathways were omitted for clarify, e.g. the direct effect from adulthood socioeconomic status (SES) to mediators

**Supplementary Figure 3**. Participant flow diagram

**Supplementary Figure 4**. Association between number of child maltreatment types and depression disorder by population subgroups

Adjusted for age, sex, ethnicity, deprivation index, and education attainment. The HRs correspond to the increase in risk for each additional types of child maltreatment.

**Supplementary Figure 5**. Association between number of child maltreatment types and anxiety disorder by population subgroups

Adjusted for age, sex, ethnicity, deprivation index, and education attainment. The HRs correspond to the increase in risk for each additional types of child maltreatment.

**Supplementary Figure 6**. Association between number of child maltreatment types and behavioural syndrome by population subgroups

 Adjusted for age, sex, ethnicity, deprivation index, and education attainment. The HRs correspond to the increase in risk for each additional types of child maltreatment.

**Supplementary figure 7.**  Association between child maltreatment and all mental health disorders including those with previous mental health diagnoses (n=68338)


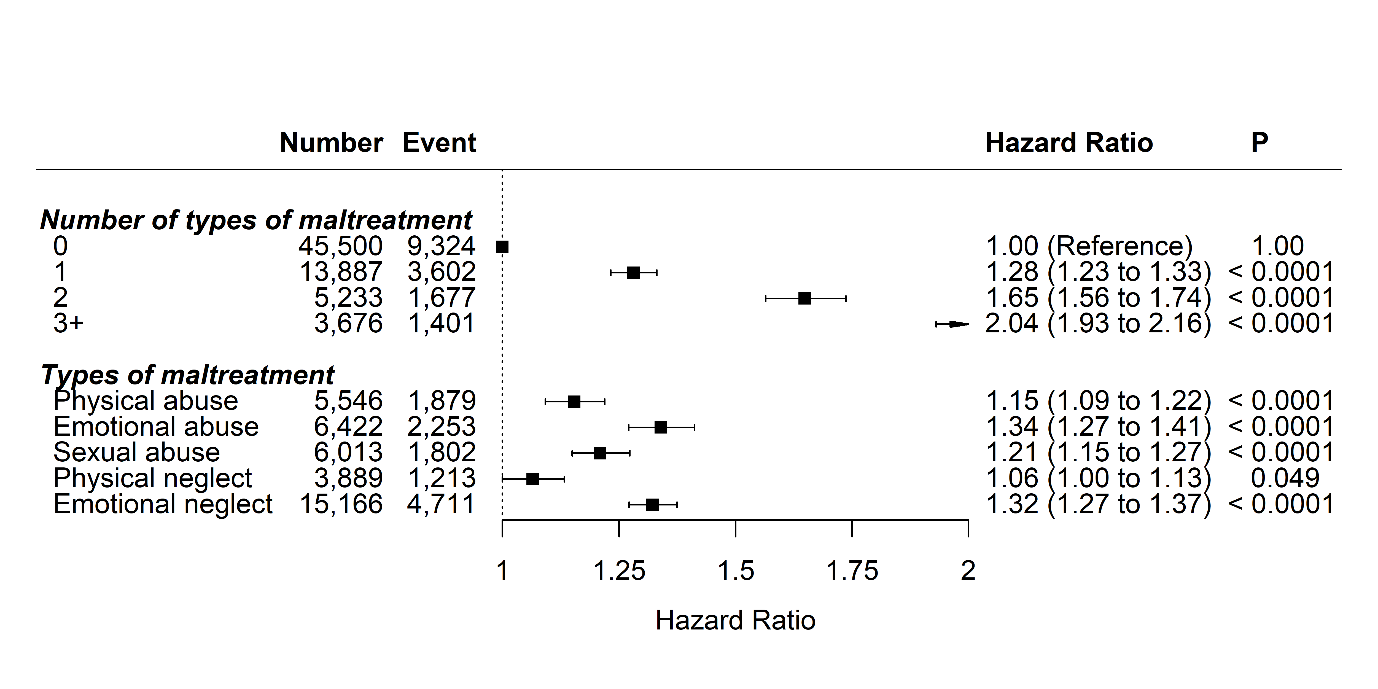


Adjusted for age, sex, ethnicity, deprivation index, and education attainment

**Supplementary figure 8.** Association between number of maltreatment types and mental outcomes disorders including those with previous mental health diagnoses (n=68338)


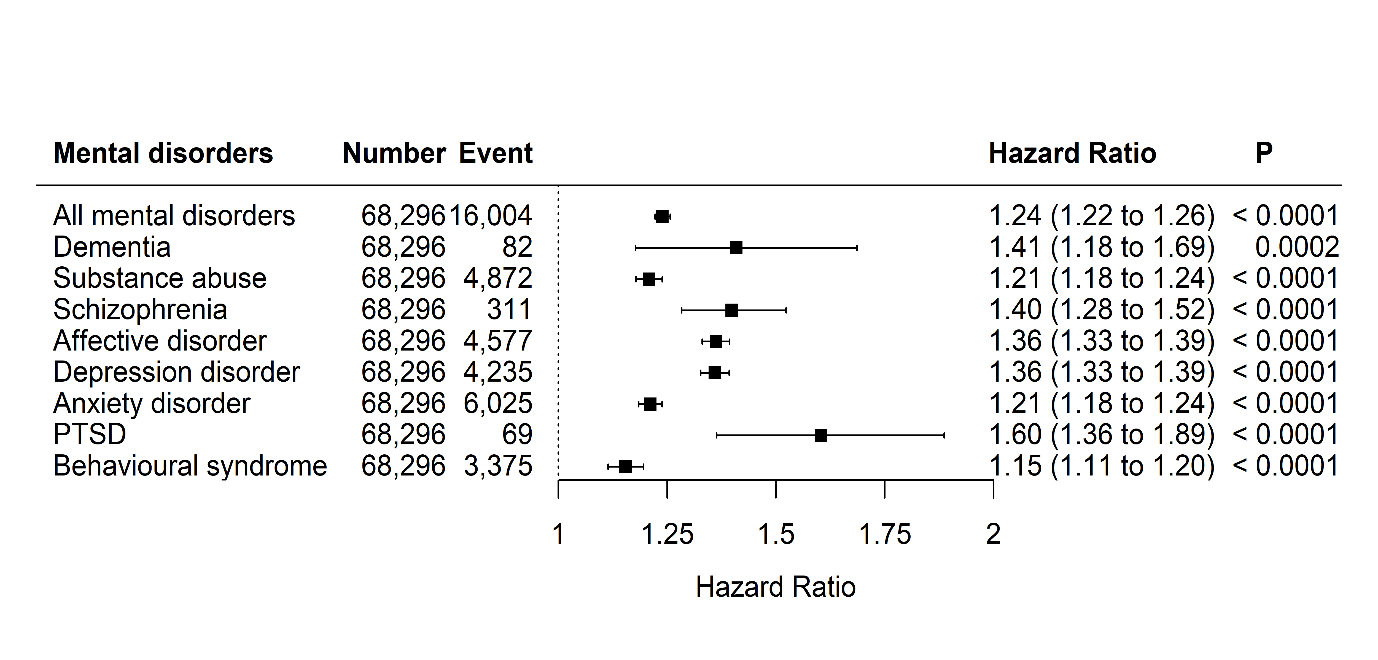
Adjusted for age, sex, ethnicity, deprivation index, and education attainment. The HRs correspond to the increase in risk for each additional types of child maltreatment.

**Supplementary figure 9.**  Association between number of child maltreatment types and all mental disorders by subgroups disorders including those with previous mental health diagnoses (n=68338)


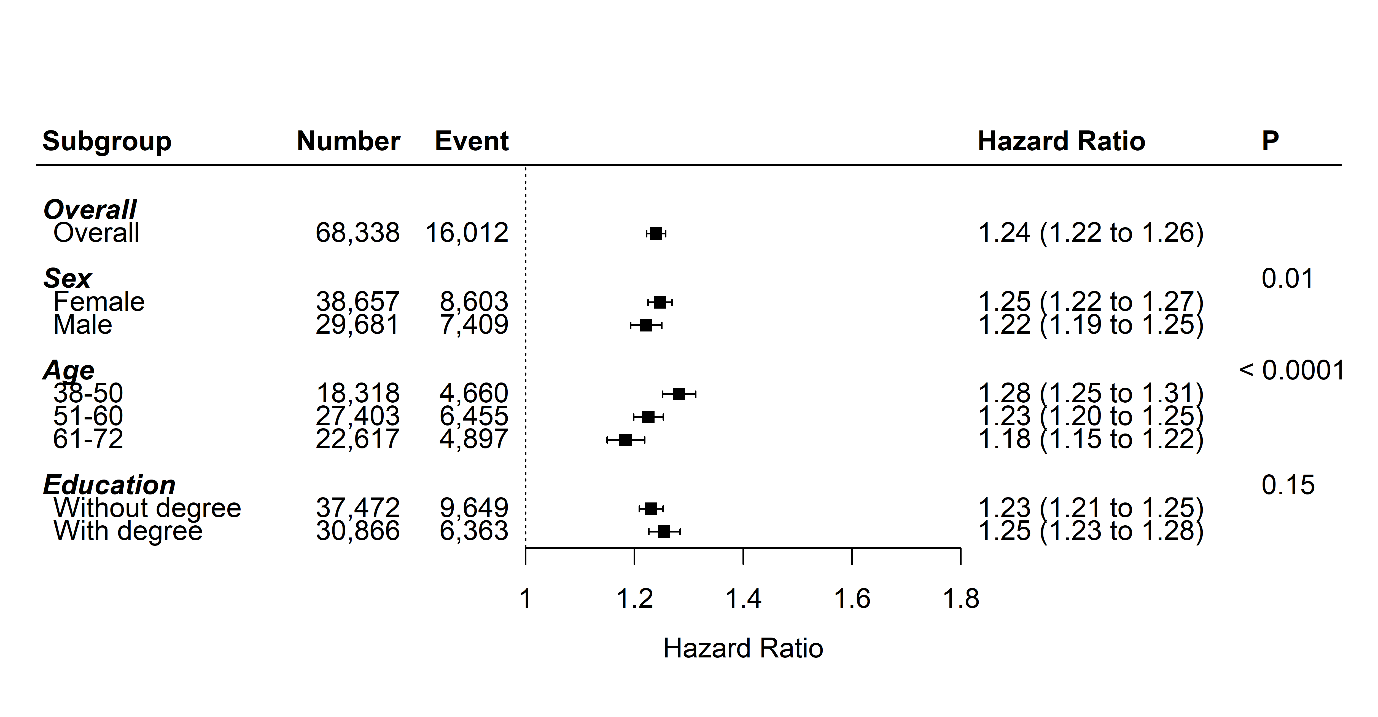


Adjusted for age, sex, ethnicity, deprivation index, and education attainment. The HRs correspond to the increase in risk for each additional types of child maltreatment.
